# Sex-Specific Analysis of Mid-Term Outcomes of Atherectomy-Assisted Endovascular Treatment in Severe Peripheral Arterial Disease

**DOI:** 10.3390/jcm13113235

**Published:** 2024-05-30

**Authors:** Konstantinos Avranas, Apostolos G. Pitoulias, Gergana T. Taneva, Efthymios Beropoulis, Konstantinos P. Donas

**Affiliations:** Rhein Main Vascular Center, Department of Vascular and Endovascular Surgery, Asklepios Clinics Langen, Paulinen Wiesbaden, 63225 Langen, Germany; avranaskon@gmail.com (K.A.); dr.gtaneva@gmail.com (G.T.T.); eberopoulis@gmail.com (E.B.); konstantinos.donas@googlemail.com (K.P.D.)

**Keywords:** rotational atherectomy, atherectomy-assisted drug-eluting angioplasty, femoropopliteal lesions, peripheral artery disease, sex-specific outcomes

## Abstract

**Background:** Endovascular treatment of lower-extremity peripheral disease (PAD) is associated with higher complication rates and suboptimal outcomes in women. Atherectomy has shown favourable outcomes in calcified lesions, minimising the incidence of stent placement caused by recoil or flow-limiting dissection. To date, there are no published mid-term outcomes evaluating the performance of atherectomy differentiated by sex. This study aims to evaluate sex-specific outcomes and prognostic factors affecting the results of atherectomy-assisted endovascular treatment in severe PAD. **Methods:** A retrospective analysis was conducted at a single centre in Germany, initiated by physicians and not sponsored by industry, on patients presenting with Rutherford categories ranging from III to V and featuring de novo occlusive or stenotic lesions of the superficial femoral (SFA) and/or popliteal arteries. The intervention involved rotational atherectomy-assisted angioplasty utilising the Jetstream (Boston, US^®^) device. The point of interest of this study was postinterventional clinical improvement as well as mid-term outcomes, including primary patency, over a targeted 2-year follow-up period. Statistical analysis utilised Cox regression (survival analysis) to calculate hazard ratios according to sex category. Comparative survival analysis was performed using the log-rank test and visually represented through Kaplan–Meier curves. Risk factors associated with absence of clinical improvement were examined across both sex groups utilising the chi-square or Fisher exact test, as appropriate. **Results:** A total of 98 patients (103 limbs) were initially included, with >75% having moderate-to-severe lesion calcification (>50%). A total of 84 patients (97 limbs, 62 male and 35 female) proceeded to a 2-year follow-up (mean 16.4 months for males and 16.1 for females) after a successful index procedure. Age distribution, Rutherford class, diabetes, chronic kidney disease (CKD), target vessel, lesion type, and length were balanced among both groups. Similar primary patency rates, of 89% among female and 91% among male limbs, were observed (*p* = 0.471). Female patients exhibited a lower rate of clinical improvement based on the Rutherford scale in comparison to males (80.6% vs. 94.5%, *p* = 0.048). CDK was the only significant prognostic factor across pooled data (odds ratio for CKD: 15.15, *p* < 0.001). **Conclusions:** Rotational atherectomy showed comparably high rates of mid-term primary patency, with low rates of bailout stent placement. These findings highlight the beneficial use of atherectomy in female patients who are per se at risk for higher rates of complications during and after endovascular interventions.

## 1. Introduction

The management of atherosclerotic femoral–popliteal disease poses persistent challenges in clinical practice. The presence of calcium deposits further complicates treatment interventions, particularly with stents, as they are susceptible to extrinsic compression, suboptimal expansion, and long-term fracture. Additionally, atheromatous lesions containing thrombotic elements heighten the risk of peripheral embolisation during an intervention [1].

Atherectomy procedures play a pivotal role in optimising vessel preparation for subsequent angioplasty by effectively debulking plaque burden while mitigating the risk of vessel recoil and dissection, thus reducing the need for subsequent stent placement. Despite the increasing adoption of atherectomy techniques, the diversity of available platforms and devices obscures the comparative performance of each device in the context of severe peripheral arterial disease (PAD) affecting the lower extremities [2].

The Multicentric National Registry trial on the use of rotational atherectomy in femoral–popliteal occlusive atherosclerotic disease (MORPHEAS) is the first study to compare results from four individual vascular centres which demonstrated reproducible between-hospital outcomes regarding the use of rotational atherectomy with the Jetstream Atherectomy device XC (Pathway Medical Technologies, Inc., Redmond, WA, USA) for the treatment of femoropopliteal occlusive disease [3]. At 12 months’ follow-up, freedom from target lesion revascularisation in complex lesions of the superficial femoral artery (SFA) and infrainguinal arterial disease appears to be between 80 and 90% in different studies [4,5,6]. Teneva et. al. revealed high rates of primary patency of 97% at 12 and 83 at 24 months [7].

Recent studies have shown that rotational atherectomy can be beneficial for the treatment of severe PAD even in fine and small-calibre below-the-knee (BTK) arteries, with promising results and with low rates of bailout stenting as well as target lesion revascularisation (TLR) [8,9]. Additionally, Pitoulias et al. compared the early results of plain old balloon angioplasty (POBA) and atherectomy-assisted angioplasty in chronic limb-threatening ischemia (CLTI) and showed favourable outcomes with lower amputation rates and higher primary patency for the atherectomy interventions [10].

Although the effectiveness and safety of rotational atherectomy has been proven to be adequate and its use feasible for the treatment of both suprapopliteal and infrapopliteal PAD disease, its application in the smaller and more delicate arteries of women has not been fully investigated.

Regarding sex-specific outcomes, female patients undergoing interventions for the treatment of peripheral vascular disease exhibited increased rates of complications including dissection, distal embolisation, above-the-knee amputation, and even in-hospital mortality. Interestingly, women were less inclined to undergo atherectomy as vessel preparation but were more likely to undergo reintervention [11,12]. The current literature is still lacking subgroup analyses, especially sex-based, on patients with highly calcified lesions. More sex-oriented studies are required for the establishment of more appropriate and optimal therapies for the treatment of heavily calcified peripheral artery disease in women.

Sex-specific outcomes following vessel preparation with intravascular lithotripsy demonstrated favourable acute safety profiles and achieved low percentages of residual stenosis in both sexes. However, these findings were limited by the absence of subsequent follow-up assessments [12].

Motivated by the promising results of intravascular lithotripsy in treating severe PAD in the female population as well as the established benefits of plaque debulking and vessel preparation with rotational atherectomy and the notable absence of data on how sex influences the outcomes of rotational atherectomy-assisted angioplasty in women, our study aims to assess sex-specific outcomes of RA for the treatment of symptomatic heavily calcified PAD of the lower extremities.

## 2. Methods

This retrospective analysis, conducted at a single centre in Germany, was initiated by physicians and did not receive sponsorship or external funding from industry. This study utilised prospectively collected data with subsequent anonymisation, adhering to the guidelines set forth by the institutional research committee and ensuring compliance with the principles outlined in the Declaration of Helsinki. Informed consent was obtained from all subjects involved in this study. This study was approved by the local ethics committee, the regional medical association of Hesse, in 2023 (ethical approval number: 2023-3323-evBO).

### 2.1. Selection Criterion

Patients with PAD in Rutherford categories ranging from III to V who underwent endovascular interventions by employing atherectomy-assisted angioplasty were considered for inclusion in this study. Angiographic eligibility necessitated the presence of de novo occlusive or stenotic lesion disease in the superficial femoral (SFA) and/or popliteal arteries, with a reference vessel diameter between 4 and 7 mm. Lesions were classified according to the TransAtlantic Inter-Society Consensus (TASC) II [3,13] classification for femoropopliteal lesions. The Fanelli score was also employed for the classification of lesions by their calcification grade [14].

Exclusion criteria encompassed prior ipsilateral bypass surgery, systemic coagulopathy, end-stage renal disease requiring haemodialysis, life expectancy of less than 12 months, aspirin, clopidogrel, and/or heparin intolerance, as well as any other factors hindering treatment or compliance with follow-up. Atherectomy was not pursued in cases of subintimal recanalisation. The absence of a patent origin of the SFA (stump) also led to exclusion from the study. According to protocol, femoropopliteal bypass surgery would be prioritised as the initial treatment approach for patients, thereby avoiding retrograde vessel manipulation and subsequent attempts.

### 2.2. Definitions

Severe peripheral artery disease (PAD) was characterised by the clinical manifestation of Rutherford categories III to V. Technical success in the atherectomy-assisted angioplasty procedure was delineated by the effective treatment of lesions, resulting in residual stenosis of less than 30%. Both operations were performed by surgeons with varying levels of experience.

To our knowledge, there is no universally accepted definition of an experienced surgeon. For the purposes of this study, an experienced surgeon was defined as having more than seven years of experience as a consultant in vascular surgery and holding independent certification from the national society of vascular surgery as an endovascular specialist.

### 2.3. Endpoints

The primary endpoint of this study included the assessment of sex-specific primary patency rates confirmed by duplex sonography. A secondary endpoint was postinterventional clinical improvement based on the Rutherford scale, which comprised either an increase in walking distance or relief of resting pain in case of Rutherford category V. In case of absence of clinical improvement, patent endovascular reconstruction was confirmed by duplex sonography or computed tomography angiography. Reintervention in the reconstruction side due to occlusion or high-degree > 70% stenosis, in combination with impairment of free walking distance of one or more category in the Rutherford scale, were defined as clinical-driven targets of lesion revascularisation (CD-TLR).

### 2.4. Technique

All interventions were conducted utilising a C-arm (Ziehm Vision RFD Hybrid Edition) in the supine position. Preoperative assessment for the feasibility of ipsilateral or contralateral percutaneous approaches involved duplex ultrasound or CTA to exclude severe stenosis or circumferential calcification of the common femoral artery. According to the local protocol, an ipsilateral antegrade approach was exclusively pursued if the superficial femoral artery (SFA) segment remained patent, specifically when the diseased segment was distal to the SFA or within the popliteal arteries. Alternatively, a contralateral approach with crossover technique was employed.

A 0.035′′ support guidewire facilitated the advancement of a long 7F introducer sheath (Flexor; Cook, Bloomington, IN, USA) just proximal to the treatment segment. Subsequently, diagnostic angiography was performed, followed by lesion crossing using either an 0.018 stiff or 0.014 chronic total occlusion (CTO) guidewire over a support catheter. The utilisation of filters in the third segment of the popliteal artery varied based on the operator’s preferences [3]. Plaque debulking was accomplished through rotational atherectomy using the Jetstream System as per previously described specifications. The device was consistently advanced at a steady rate of 1 mm/s, with intermittent activation pauses every 40 s at at least 20 s intervals.

Post-rotational atherectomy plaque debulking, drug-eluting balloon (DEB) angioplasty was executed, followed by a final angiography to assess treatment adequacy and identify potential complications such as recoil or flow-limiting dissection as well as evaluate tibial vessel runoff. In cases of recoil, a prolonged angioplasty (5 min) was repeated. A stent was deployed with an estimated oversizing of 1:1 if flow-limiting dissection occurred, resulting in narrowing of the vessel lumen more than 30%. Patients not previously prescribed were initiated postoperatively on Acetylsalicylic Acid (ASA) and statins. For patients receiving adjunctive stenting, dual antiplatelet therapy was administered with clopidogrel 75 mg daily for 8 weeks, followed by lifelong monotherapy with ASA [3].

### 2.5. Follow-Up

The follow-up of patients was aimed at a 2-year duration through a combination of telephone contact and clinical examinations on an ambulatory basis. Patients were contacted periodically to assess their clinical status and inquire about any adverse events. Additionally, ambulatory clinical examinations including diagnostic imaging studies as deemed necessary were scheduled at regular intervals during the follow-up period to evaluate patient outcomes, monitor treatment efficacy, and detect any potential complications.

### 2.6. Statistics

Continuous data following a normal distribution are summarised using means and standard deviations (SDs), while non-normally distributed continuous data are presented as medians with interquartile ranges. To evaluate the normality of continuous data, the Shapiro–Wilk test was conducted. Categorical variables are expressed as counts and percentages. For the distribution of binary results, the chi square or Fisher’s exact test were conducted, as appropriate. Cox regression analysis, specifically proportional hazards regression, was employed to investigate the association between the predictor variable (sex category) and the time until specific event occurred (loss of primary patency). Survival curves were compared using the log-rank test, and graphical representation was facilitated through Kaplan–Meier survival curves [15]. Statistical significance was determined at the *p* < 0.05 level. Data analyses were conducted using SPSS 28 (Statistical Package for the Social Sciences, Inc., Chicago, IL, USA).

## 3. Results

### 3.1. Demographic Data

A total of 98 patients were enrolled, whereas 103 limbs were treated in our cohort, with the majority being males (62%). Table 1 depicts details on the demographics and comorbidities. Male patients were slightly younger (74.7 vs. 77.7 median age, *p* = 0.091) with higher prevalence of concomitant coronary artery disease (CAD). Over 80% of male patients were on antiplatelet or anticoagulation therapy, whereas this percentage was 65% for females, probably due to the lower prevalence of CAD (50% in male vs. 17.9% in female patients, *p* = 0.001), as shown in Table 1 and Table 2.

### 3.2. Operative Data and Results

Table 3 depicts details on operative data and results. Vascular access sites were comparable across both groups (81.3% crossover in men and 84.6% in women) and technical success was achieved in 95.3% and 94.9% of cases, respectively. The mean operative time was 107.6 (±25.3) min for male limbs and 102.3 (±22.2) min for females; 68.8% of the former and 61.5% of the latter were intervened in by experienced operators (*p* = 0.454). Table 4 depicts the angiographic characteristics of the lesions. No statistical difference in the morphology and calcification grade of the lesions was found between the two groups.

An experience demanding vascular surgery with below-the-knee bypass using a reversed vein as graft material was needed in one male patient due to extensive occlusion caused by heparin-induced thrombocytopenia. Two patients (one of which was female) presented with lifestyle-limiting claudication and after a failed revascularisation attempt, they were treated conservatively. In one case, an endovascular reintervention was required to maintain primary target lesion patency. Access site complications were documented in six patients, three of them being male and three female (4.7% and 7.7% respectively, *p* = 0.671), and manifested as a haematoma or wound infection. In one male patient, an occlusion of the contralateral femoral artery due to percutaneous closure device (Angio-Seal, St. Jude Medical, Saint Paul, MN, USA) was observed and managed by surgical thrombectomy. As Table 5 and Table 6 depict, there was no difference between experienced and young operators regarding technical aspects, including technical success.

### 3.3. Clinical Improvement

Clinical improvement with amelioration of Rutherford category was achieved postprocedurally in 52 males (94.5%) and 25 females (80.6%) (*p* = 0.044) with given reconstruction patency (examined as mentioned above). In cumulative data, the experience level of the operator as well as the lesion length do not influence the outcome regarding clinical improvement. On the contrary, CKD appears to be the only independent risk factor (odds ratio for CKD: 15.15 (95% CI: 3.2–58.8), as summarised in Table 7.

### 3.4. Follow-Up Data

Follow up duration was 16.45 (±8.02) months for male and 16.14 (±6.88) months for female patients (*p* = 0.897).

### 3.5. Patency Rates

**Figure 1:**
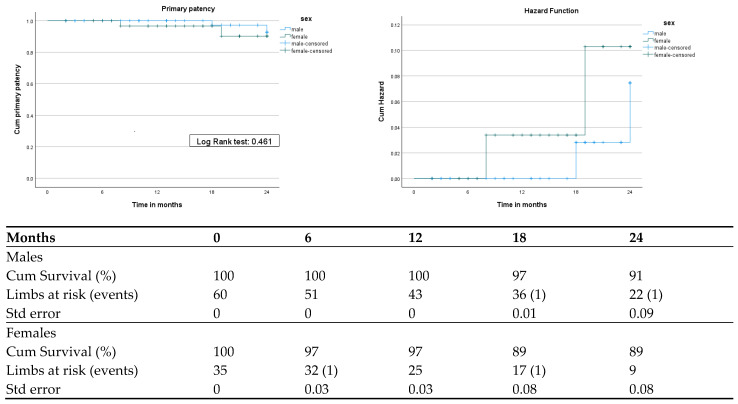
Kaplan–Meier and cumulative hazard curves of primary patency.

Primary patency rates were 91% for male and 89% for female limbs after 24 months. The log-rank test indicated no significant differences between the two groups (*p* = 0.461). The Cox regression model for primary patency revealed a hazard ratio (HR) (female/male) of 2.061 (95% CI: 0.289–14.708), *p* = 0.471, with mean times of sustained primary patency 23.83 (95% CI: 23.37–224.2) months for males and 23.14 (95% CI: 21.9–24.3) months for females (Figure 1).

## 4. Discussion

### 4.1. Sex Category and Mid-Term Outcomes after Atherectomy

This paper serves as a post hoc analysis of mid-term outcomes in patients treated with rotational atherectomy on grounds of severe peripheral femoropopliteal arterial disease [7], scrutinising the effect of sex on primary patency and postoperative clinical improvement with amelioration of Rutherford category, to our knowledge for the first time in the literature. We identified similar performance in both sexes independent of operator experience, with high rates of technical success (95.3% for males and 94.9% for females) and low incidence of additional stenting (6.3% and 5.1%, respectively). Women exhibited a higher necessity for additional covered stents (4.8% vs. 17.9%, respectively), mainly due to a higher burden of mixed with a domination of softer thrombotic material. It is also important to mention here that the Jetstream device also has indications for the treatment of soft plaques due to its aspiration thrombectomy capacity parallel to its atherectomy function.

During the follow-up period, comparable rates of primary patency at 24 months were observed between the sexes.

The disproportionately low presentation of women and at a relatively higher age in our series seems to be comparable to surveillance data in Germany between 2009 and 2018, where PAD was higher in male patients, with only a female minority of 37.1% presenting to a vascular specialist both later in the course of the disease and with a more advanced and severe clinical stage [16].

According to pooled data of the Korean Vascular Intervention Society Endovascular Therapy in Lower Limb Artery Diseases (K-VIS ELLA Registry) of over 3000 patients (550 women included) following endovascular treatment for symptomatic PAD, women exhibited higher rates of complex lesions than men and higher rates of major amputations, procedural complications, such as death and myocardial infarction, as well as limb-specific adverse events, including reinterventions and minor amputations. The authors of the aforementioned study attributed those unfavourable events and complications to the smaller vessel diameter and higher prevalence of multilevel disease observed in women [17]. It should be noted that in this registry, the treatment option included all endovascular techniques. The choice of technique was left to the operator’s discretion, without specifying whether atherectomy or other vessel preparation methods were used prior to the planned percutaneous transluminal angioplasty (PTA).

Nagpal et al. in their sex-specified sub-analysis based on the Disrupt PAD III observational study demonstrated that despite presenting in a worse clinical stage, with lower ankle–branchial index (ABI), smaller vessel diameter, and more calcified higher-grade stenosis, women who underwent vessel preparation with IVL had statistically significantly lower rates of residual stenosis compared to their male counterparts. These postprocedural data after vessel preparation with intravascular lithotripsy which revealed favourable acute effectiveness in both sexes despite the presence of smaller reference vessels in women underline the importance of vessel preparation even in finer and more delicate arteries [12].

On the contrary, Lee et al. in a pooled analysis of the CONFIRM I, II, and III registries on the possible influence of the sex category on the outcomes of orbital atherectomy, which included more than 3000 patients (1261 of which female), revealed higher rates of dissections (13.3% vs. 9.9%; *p* < 0.001) and distal embolisations (2.8% vs. 1.9%, *p* = 0.07) in women [18]. It is worth mentioning that women presented in a worse clinical stage, with 45% of them exhibiting critical limb-threatening ischemia (CLTI), and were significantly older in comparison with the enrolled males. However, only 1.6% of the documented dissections in women were flow-limiting (versus 1.4% in male patients).

Contradicting the two aforementioned studies, the calcification grade and the length of lesions as well as the prevalence of stenosis or occlusion were similar between the two groups in our study, showcasing comparable vessel quality in both sexes.

Women exhibited higher rates of absence of clinical improvement compared to men, at 19.4% versus 5.5%, respectively (*p* = 0.048), probably due to worse clinical stage at the day of admission to the hospital. In detail, 60% of women had signs of CLTI, while only 48% of the male patients had ischemic rest pain or tissue loss, consolidating the current literature.

These findings mirror the sex disparities of the female and male biology, as women exhibit a higher risk of vessel thrombosis [19]. Factors such as advanced age at presentation, smaller arterial diameters, comorbidities, and later diagnosis with advanced disease burden are postulated to explain higher complication rates and worse outcomes in women undergoing peripheral vascular interventions.

Growing evidence indicates that sex chromosomes play a significant role in the course of atherosclerosis. 

One notable aspect of endothelial dysfunction involves compromised macro- and microvascular endothelium-dependent vasodilation, partly due to diminished nitric oxide availability. While men typically experience measurable impairment in endothelial function in their fourth decade, women tend to experience this impairment approximately ten years later, often coinciding with menopause. Subsequently, women may exhibit an acceleration in endothelial dysfunction, leading to a level of impairment similar to that seen in men. The differential risk of PAD between sexes may be influenced by metabolic and inflammatory pathways in women, whereas in men, risk factors may be more associated with coagulation abnormalities and other factors [20].

### 4.2. Chronic Kidney Disease and Peripheral Arterial Disease

CKD plays a pivotal role in cardiovascular disease, with strong correlation to microvascular disfunction [21], being extensively studied in the neurologic field with characteristic MRI findings due to small-vessel disease [22].

In the field of PAD, individuals with CKD experience significantly worse outcomes after interventions despite comparable primary patency rates. Multiple studies have strongly indicated worse outcomes after revascularisation, including limb salvage with higher rates of amputation, despite similar graft patency.

In cases with CKD patients who underwent infrapopliteal bypass, patients on haemodialysis had an almost 5-fold higher risk of limb loss compared to their counterparts with CKD who did not require haemodialysis and significantly lower rates of 5-year amputation-free survival (43.6% vs. 78.8%, *p* = 0.0033). This increased risk of amputation occurred despite equivalent rates of revascularisation of the target limb (12.4% vs. 12.2%) [23]. In another prospective study, patients who underwent elective infrainguinal bypass for CLTI and were on dialysis had a 3.7-fold increased risk of clinical failure, being defined as amputation or persistence or worsening of ischemic symptoms despite graft patency, compared to patients with normal renal function [24]. Patel et al. interestingly reported that the presence of end-stage CKD in patients undergoing percutaneous endovascular interventions increased the risk of late amputation despite having patent bypass grafts, whereas freedom from reinterventions remained similar after 1 year [25].

The above findings are attributable to microvascular rarefaction, resulting in impaired microcirculation in various vascular beds, including skeletal muscles, in CKD patients and animal models, which may contribute to this phenomenon [26]. In summary, CDK is proven to be an undermining factor in the clinical improvement of patients and lower-extremity longevity, despite the high rates of technically successful revascularisation and patency.

## 5. Limitations

This study is limited by its observational nature. Only 38% of limbs at risk were female, with higher age and lower prevalence of coronary arterial disease and dyslipidaemia. While our analysis offers valuable insights, this study’s scope and size may not fully encompass the complexities of the phenomenon. Larger confirmatory studies involving patient matching after the involvement of multiple centres are essential. These future endeavours will validate and enhance the findings, fostering a deeper understanding of the subject.

## 6. Conclusions

The use of rotational atherectomy-assisted angioplasty for severe femoropopliteal disease showed high rates of primary patency in both sex categories after a two-year follow-up period. The high rates of technical success without the need of additional stent implantation in the majority of patients, combined with the high mid-term primary patency rates, highlight the effectiveness and safety of rotational atherectomy in both sexes. This consolidates rotational atherectomy as a valid plaque debulking and vessel preparation technique, especially among female patients with more challenging vascular pathology and anatomy.

Advanced chronic kidney disease appears to be an independent risk factor throughout both sex categories.

Nonetheless, long-term follow-up with comparative analysis across larger patient cohorts is essential to validate and strengthen the above findings.

## Figures and Tables

**Table 1:** Baseline characteristics.

Characteristic	Males (N = 64)	Females (N = 39)	*p* Value
Age	74.75 (±10.7)	77.72 (±6.8)	0.091
Age over 80	32 (50%)	18 (46.2%)	0.705
Smoking history	40 (62.5%)	17 (43.6%)	0.061
Hypertension	51 (79.7%)	34 (87.2%)	0.331
Dyslipidaemia	46 (71.9%)	18 (46.2%)	0.009
Diabetes	26 (40.6%)	16 (41%)	0.968
Coronary artery disease	32 (50%)	7 (17.9%)	0.001
Chronic kidney disease	10 (15.6%)	11 (28.2%)	0.124
Rutherford class			0.592
I	0	0	
II	0	0	
III	33 (51.6%)	15 (38.5%)	
IV	20 (31.3%)	14 (35.9%)	
V	11 (17.2%)	10 (25.7%)	

**Table 2:** Preinterventional antithrombotic and anticoagulation therapy.

Medication	Males (N = 64)	Females (N = 39)	*p* Value
Aspirin	39 (60.9%)	16 (41%)	0.088
Clopidogrel	3 (4.7%)	0	0.278
Vitamin K antagonist	5 (7.8%)	2 (5.1%)	0.707
Direct anticoagulants	7 (10.9%)	7 (17.9%)	0.379

**Table 3:** Operative characteristics.

	Males (N = 64)	Females (N = 39)	*p* Value
Experienced operator	44 (68.8%)	24 (61.5%)	0.454
Vascular access			0.663
Antegrade femoral	12 (18.8%)	6 (15.4%)	
Crossover femoral	52 (81.3%)	33 (84.6%)	
Additional stent	4 (6.3%)	2 (5.1%)	0.582
Additional stent-graft	3 (4.8%)	7 (17.9%)	0.035
Technical success (residual stenosis < 30%)	61 (95.3%)	33 (94.9%)	0.92
Postprocedural clinical improvement	52 (94.5%)	25 (80.6%)	0.044
Intraprocedural complications	6 (9.4%)	3 (7.9%)	0.799
Access site complications	3 (4.7%)	3 (7.7%)	0.671
Operation duration (min)	107.6 (±25.3)	102.3 (±22.2)	0.283

**Table 4:** Angiographic characteristics of the target lesion.

	Males (N = 64)	Females (N = 39)	*p* Value
Lesion Type			**0.735**
Stenosis	18 (28.1%)	11 (28.2%)	
Occlusion	45 (70.3%)	28 (71.8%)	
Target vessel			0.595
SFA	34 (53.1%)	25 (64.1%)	
Target vessel SFA-PI	16 (25%)	8 (20%.5)	
Target vessel PII-PIII	9 (14.1%)	5 (12.8%)	
Target vessel BTK	5 (7.8%)	1 (2.6%)	
No of run-off vessels 0			0.527
0	0	1 (2.6%)	
1	13 (20.3%)	6 (15.4%)	
2	17 (26.6%)	9 (23.1%)	
3	34 (53.14%)	23 (59%)	
Calcification grade			0.387
25%	2 (3.1%)	1 (2.6%)	
25–50%	7 (10.9%)	9 (23.1%)	
50–75%	32 (50%)	15 (38.5%)	
Over 75%	23 (35.9%)	14 (35.9%)	
Lesion length (mm)	97.4 (±59.6)	101.8 (±64.5)	0.727

SFA: superficial femoral artery, P: popliteal, BTK: below-the-knee.

**Table 5:** Technical aspects in male patients according to operator groups.

Technical Aspects	Experienced Operators	Young Operators	*p* Value
Technical success (residual stenosis < 30%)	42/44 (95.5%)	19/20 (95%)	0.936
Intraprocedural complications	6/44 (13.6%)	0/20 (0%)	0.165
Access site complications	2/44 (4.5%)	1/20 (5%)	0.936

**Table 6:** Technical aspects in female patients according to operator groups.

	Experienced Operators	Young Operators	*p* Value
Technical success (residual stenosis < 30%)	22/24 (91.7%)	15/15 (100%)	0.251
Intraprocedural complications	3/24 (12.5%)	0/15 (0%)	0.283
Access site complications	1/24 (4.2%)	2/15 (13.3%)	0.547

**Table 7:** Risk factors for the absence of clinical improvement on the Rutherford scale (cumulative data).

	Improvement (77)	Non-Improvement (9)	*p* Value
Experienced operators	51 (66.2%)	6 (66.6%)	0.646
Smoking history	30 (38.9%)	3 (33.3%)	9.523
History of diabetes	45 (58.4%)	6 (66.6%)	0.733
CAD	43 (55.8%)	7 (77.7%)	0.293
CKD	68 (88.3%)	3 (33.3%)	<0.001
Lesion length	98.1 (±61.3)	85.7 (±41)	0.47

CAD: coronary arterial disease, CKD: chronic kidney disease.

## Data Availability

Data are available on request from the first two co-authors (K.A. and A.P.).
